# Phase and power modulations on the amplitude of TMS-induced motor evoked potentials

**DOI:** 10.1371/journal.pone.0255815

**Published:** 2021-09-16

**Authors:** Lukas Schilberg, Sanne Ten Oever, Teresa Schuhmann, Alexander T. Sack

**Affiliations:** 1 Section Brain Stimulation and Cognition, Department of Cognitive Neuroscience, Faculty of Psychology and Neuroscience, Maastricht University, Maastricht, The Netherlands; 2 Language and Computation in Neural Systems Group, Max Planck Institute for Psycholinguistics, Nijmegen, The Netherlands; 3 Donders Institute for Neuroimaging, Radboud University, Nijmegen, The Netherlands; 4 Maastricht Brain Imaging Centre (MBIC), Maastricht University, Maastricht, The Netherlands; 5 Faculty of Psychology and Neuroscience, Faculty of Health, Medicine and Life Sciences, Centre for Integrative Neuroscience, Maastricht University, Maastricht, The Netherlands; 6 School for Mental Health and Neuroscience (MHeNS), Department of Psychiatry and Neuropsychology, Faculty of Health, Medicine and Life Sciences, Maastricht University Medical Centre, Maastricht, The Netherlands; Universita degli Studi di Trento, ITALY

## Abstract

The evaluation of transcranial magnetic stimulation (TMS)-induced motor evoked potentials (MEPs) promises valuable information about fundamental brain related mechanisms and may serve as a diagnostic tool for clinical monitoring of therapeutic progress or surgery procedures. However, reports about spontaneous fluctuations of MEP amplitudes causing high intra-individual variability have led to increased concerns about the reliability of this measure. One possible cause for high variability of MEPs could be neuronal oscillatory activity, which reflects fluctuations of membrane potentials that systematically increase and decrease the excitability of neuronal networks. Here, we investigate the dependence of MEP amplitude on oscillation power and phase by combining the application of single pulse TMS over the primary motor cortex with concurrent recordings of electromyography and electroencephalography. Our results show that MEP amplitude is correlated to alpha phase, alpha power as well as beta phase. These findings may help explain corticospinal excitability fluctuations by highlighting the modulatory effect of alpha and beta phase on MEPs. In the future, controlling for such a causal relationship may allow for the development of new protocols, improve this method as a (diagnostic) tool and increase the specificity and efficacy of general TMS applications.

## Introduction

Transcranial magnetic stimulation (TMS) allows for a non-invasive investigation of corticospinal excitability. TMS-induced motor evoked potentials (MEPs) represent the excitability of the corticospinal tract, at which nerve fibers connect the cerebral motor cortex with the spinal cord for voluntary movement execution. Corticospinal excitability has become a frequently examined neurophysiological parameter in fundamental research as well as clinical studies [1, 2]. To serve as a valid and meaningful assessment tool that allows for veridical inferences about corticospinal excitability, TMS-induced MEP measures need to be stable and reliable. Whether they actually are reliable, however, has been subject for controversial debate, as concerns about high variability of trial-by-trial MEP amplitude have been a long known phenomenon [3–5] and specific biological and methodological factors contributing to the response variability of corticospinal excitability have been highlighted [6, 7].

A physiological cause for the variability of MEPs could be the influence of the naturally fluctuating state of neuronal activity in the brain at the time and location of assessment. Specifically, neuronal oscillations in the alpha and beta frequency range have been linked to sensorimotor processing [8–11]. Therefore, uncontrolled states of cortical excitability at the time and location of TMS could cause the reported variability of MEPs. Previous studies have demonstrated diverse associations of corticospinal excitability with preceding oscillation frequency power and phase. Reported findings include both the existence and absence of relationships between MEP amplitude and alpha or beta frequency power [12–18], phase [19–22] and phase-power interaction [23]. Most of these findings suggest that the dynamic state of the brain may influence the investigation of corticospinal excitability mechanisms with TMS. However, experimental paradigms and procedures differ considerably, which could explain the pronounced diversity of the reported results.

In this study, we aim to provide further insight into the relationship of the power and phase of alpha and beta oscillations with MEP amplitude as a representative measure of corticospinal excitability. For that we apply TMS over the primary motor cortex (M1) at suprathreshold intensities (120% of resting motor threshold (rMT)), while recording electroencephalography from the cortical area under stimulation. Moreover, we examine whether phase correlation with large MEPs cluster on consistent phases over participants. We hypothesize that the amplitude of induced MEPs is correlated to the power and particular phases of alpha and beta frequencies. We do not limit the phase analyses to either the peak or trough of the particular frequencies, but provide an elaborate investigation of the relationship between all frequency dependent phase angles and elevated cortical excitability.

## Materials and methods

The EEG and EMG data analyzed for this study are part of a larger TMS study on the reliability of intermittent theta burst stimulation (iTBS)-induced neuroplasticity mechanisms that has been analyzed and published separately [24]. This larger study consisted of two identical experimental sessions in which iTBS was applied to participants at rest before MEP changes to baseline were measured for sixty minutes and one control visit in which iTBS was replaced with sham-iTBS. The data for the current study was collected during that single control session, which included one block of sham-iTBS, but no other form of active TMS in addition to the experimental single TMS pulses included in the analysis. Participants did not perform any tasks during the experiment. The study was approved by the Ethics Committee of the Faculty of Psychology and Neuroscience at Maastricht University (number: 04_06_2013).

### Participants

Twenty-seven healthy participants (16 female; mean age (SD): 24.1 (3) years) were included in the study. All participants were right handed and of healthy cognition (Mini-Mental State Examination scores between 28 and 30). Participants were financially compensated for their participation.

### TMS application

TMS was applied with a MagPro X100 stimulator (MagVenture A/S, Farum, Denmark) and a figure-of-eight coil (MC-B70). Pulses were biphasic with an anterior-posterior followed by posterior-anterior current direction in the brain. The coil was placed tangentially to the scalp on top of the EEG cap (Easycap, BrainProducts, Gilching, Germany) with the handle in posterior direction orienting 45° away from the midline. Neuronavigation (Brain Voyager, Brain Innovation B.V., Maastricht, The Netherlands) was used to ensure stability of target point stimulation throughout the session. A single pulse based cortical mapping procedure was applied over left M1 to determine the hotspot for TMS-induced muscle twitches of the FDI muscle from the dominant right hand. Single pulse TMS intensity was set at 120% of the individual rMT, defined as the lowest intensity necessary to induce an MEP with a greater peak-to-peak amplitude than 0.05 mV in 50% of the trials (five out of ten). TMS was applied manually with a minimum of seven seconds between single pulses.

### EMG and EEG recording

Electromyography (EMG) signals were recorded with a Powerlab 4/34 connected to a BioAmp system (ADInstruments, Oxford, UK). EMG signals were amplified, sampled (4k/s), band-pass filtered (20-2000Hz), digitized and saved for online inspection and offline analysis with LabChart software (ADInstruments, Oxford, UK). Disposable adhesive surface electrodes (Plaquette^TM^, Technomed Europe) were attached in a belly-tendon montage over the right FDI muscle. Resting EMG signals were continuously observed to keep the peak-to-peak amplitude below 0.05 mV.

EEG was recorded with BrainAmp MR plus EEG amplifiers and BrainVision recorder (BrainProducts, Gilching, Germany). A 30-channel TMS compatible EEG-cap with Ag/AgCl electrodes (Easycap, BrainProducts, Gilching, Germany) was used with equally distributed electrode placement over the whole head (FP1, FP2, F3, F4, C3, C4, P3, P4, O1, O2, F7, F8, T7, T8, P7, P8, Fz, Cz, Pz, FC1, FC2, CP1, CP2, FC5, FC6, CP5, CP6, FT9, FT10, and left mastoid (A1)). Four additional channels for horizontal and vertical eye movement recordings were placed horizontally on the outside of both eyes and vertically below and above the left eye. The reference electrode was placed on the right mastoid (A2) and the ground electrode at AFz (10–20 EEG system). Data was recorded with a sampling rate of 2500Hz, a hardware bandpass filter of 0.1 – 1000Hz, a software low pass filter of 500Hz. Impedance was maintained under 10 kilo-ohm.

### Procedure

Before the experiment, all participants provided written informed consent. Prior to the experimental session, participants underwent a thorough safety and eligibility screening for participating in a non-invasive brain stimulation study. After a participant was screened, EEG, EMG and neuronavigation were prepared, the individual FDI hotspot was mapped and MT was determined. In addition to the single TMS pulses necessary for the preparatory procedures, participants received stimulation during eight blocks that consisted of thirty experimental single TMS pulses at 120% of individual rMT over their FDI hotspot. Single pulses within each block were administered at jittered inter-pulse-intervals of at least seven seconds.

### EMG and EEG preprocessing

EMG data were preprocessed with LabChart. Peak-to-peak MEPs and pre-pulse resting peak-to-peak EMG amplitude values were exported to Microsoft Excel. EEG data were stored with BrainVision recorder. Further analyses were performed with Matlab (MathWorks, 2017a), the FieldTrip toolbox [25], and circular statistics toolbox [26]. All trials in which no MEP was elicited were removed from the analyses (peak-to-peak MEP < 0.05 mV). To prevent any effects of pre-TMS muscle contraction on MEP amplitudes, all single trials with a pre-pulse peak-to-peak resting EMG amplitude that were further than 3 times away from the median absolute distance of every point to the median for a time window of 100ms prior to TMS, were excluded from the analysis [27]. The same criterium was held for MEP outliers and for power trials in the power analyses.

EEG data was demeaned and re-referenced to the average of all channels. Initial epoching was between -4 to 4 seconds around TMS pulse onset. Bad EEG channels as measured through visual inspection were interpolated with neighboring channels within four centimeter distance. Then, EEG was re-epoched to a time window from -2 seconds until TMS-pulse onset and resampled to 256 Hz. Eye blink correction was performed using the function ‘*scrls_regression’* within the eeglab plugin AAR (filter order 3; forgetting factor 0.999; sigma 0.01; precision 50; [28]). The main analyses was repeated using a current source density approach using the CSDtoolbox [29].

### The relationship between EEG oscillation power and phase with MEP amplitude

We extracted the logarithm of the power and phase for frequencies ranging between 2Hz to 30Hz in steps of 0.5Hz [30, 31]. A Fast Fourier transform was performed using Hanning tapers extracted over three oscillatory cycles before the TMS pulse onset (leading to different time windows and frequency resolution for different frequencies). We verified that with this analysis none of the participants displayed a phase bias (Rayleigh tests over all trials p > 0.05). For the analysis of oscillation phase, we calculated a circular-linear correlation between phase and MEP amplitude. For the analysis of power, we performed a Pearson linear correlation between power and MEP amplitude. To extract chance correlations, we calculated for each participant an estimated chance correlation by performing 1000 permutations of the correlation calculation using permuted labels for the MEP amplitudes. The number of trials included for further analyses were on average 205.44 (SD = 24.67) for the power analysis and 191.74 (SD = 24.33) for the phase analysis per participant. To get an estimation of the phase effect over time, we also extracted the Hilbert transform over the data (after applying a two-pass Butterworth filter of 8–12 Hz) and repeated the phase and power correlation analysis over time (up to -1.5 seconds prior to the TMS pulse).

The region of interest for all analyses was defined as C3 and all adjacent central electrodes (FC1, FC5, CP1, CP5) ipsilateral to the stimulation site. Correlation values at each of these channels were averaged for the initial analysis. We created a null distribution repeating the analysis using permuted labels. This null distribution reflects the expected average correlation based on chance. To statistically compare our observed values to the chance values, we compared the median of the permuted labels with the observed correlation values for power and phase separately at all frequency bins of 0.5Hz between 2Hz and 30Hz. To correct for multiple comparisons, we performed cluster statistics (clusterstatistics = ‘maxsum’, alpha = 0.05, clusteralpha = 0.05, n = 1000). Data points outside of the 95^th^ percentile of the null distribution of the monte-carlo simulation were entered in the second level cluster analysis. For the phase analysis, we report the one-sided p-value as circular correlations can only be positive. For the power analysis we report the corrected p-values. We deliberately avoid referring to μ-rhythms for the discussion of neuronal oscillations within the alpha frequency band that were measured over sensorimotor regions, because clear independence from oscillations originating in the parieto-occipital regions is not ascertained. We repeated this analysis for a different set of more posterior electrodes (O1, P3, P7, Pz, T7) to investigate the spatial specificity of this effect.

Next, we investigated whether the phase of maximal MEP size was consistent across participants. Therefore, we extracted for each participant the mean phase of the 50% highest MEPs (for all five EEG channels separately) at the alpha frequency with the highest correlation for that individual participant (limiting frequencies to frequencies within the significant cluster). Then, we performed for the circular average of the five channels a Rayleigh test to investigate the phase consistency over participants.

EEG power prior to the TMS pulse might be correlated to the activity of the EMG signal. To test this, we correlated the pre-pulse EMG activity (including all trials) with the EEG power for all tested frequency bins. Then we repeated the main power and phase analyses correcting for this correlation. For the power-MEP relation we took the residuals of the pre-pulse EMG activity and power correlation and correlated the residual power with the MEP size. For the phase-MEP relation we took the residuals of the pre-pulse EMG activity and MEP size and performed a circular-linear correlation between the phase and the residual-MEP size. Statistical tests were performed in the same manner as the main analyses.

For a final analysis we split the data in low and high power trials (median split) for each frequency bin separately and repeated the circular-linear correlations described above to investigate if the phase modulation was dependent on the oscillatory power [32]. Phase values for all figures represent the phase expected at time point ‘0’ in which ‘0’ corresponds to the peak and +/- pi to the trough.

## Results

### Effect of neuronal oscillation power on MEP amplitude

**Fig 1:**
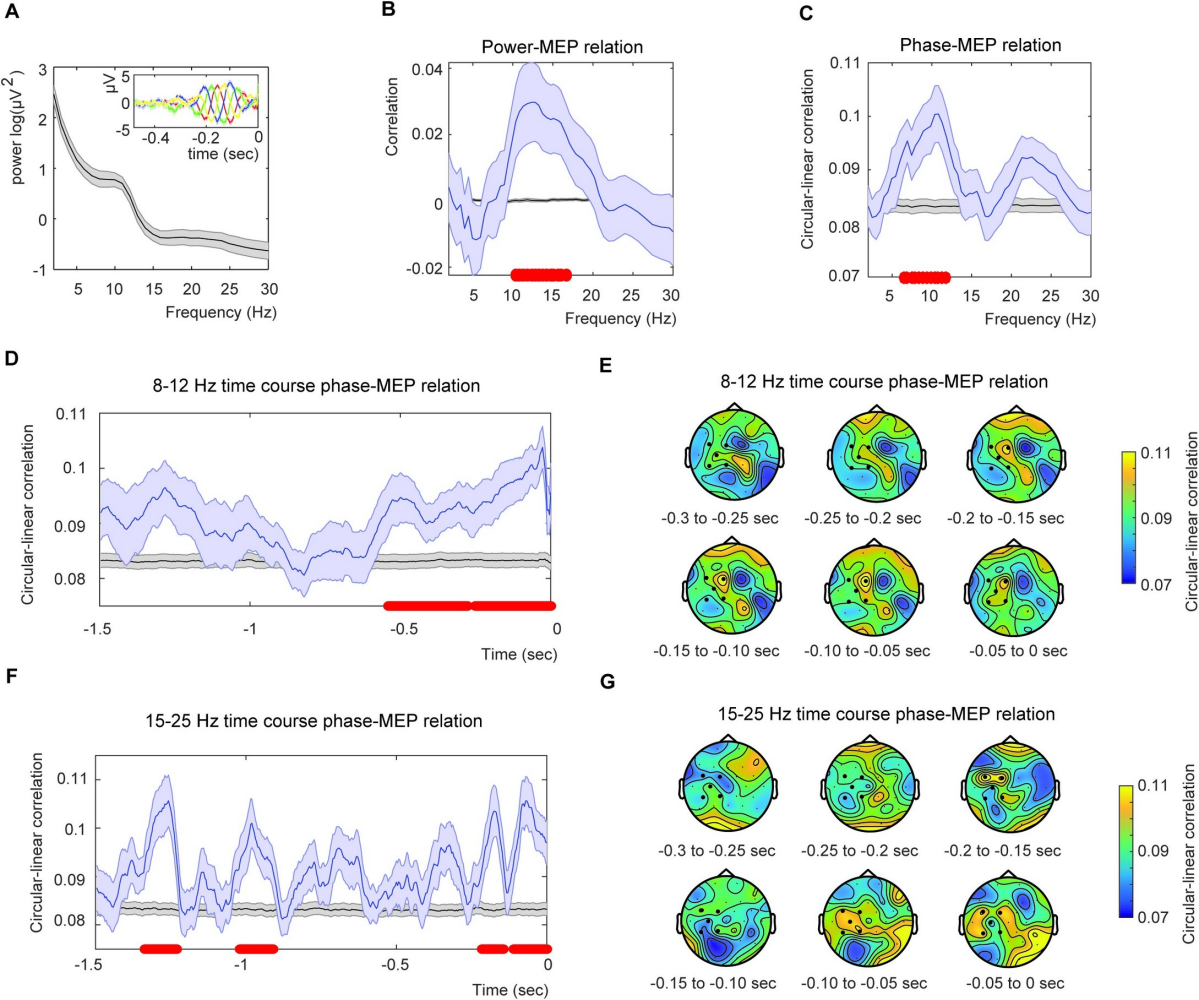
Results for the power and phase analysis. A) Mean power averaged from the EEG channels C3, FC1, FC5, CP1 and CP5. The inset shows the (unfiltered) ERPs for four different equally spaced phase bins in channel CP1 for alpha. This figure demonstrates that there is no phase bias in our estimation. B) Averaged power-MEP correlation for the five selected channels (blue) and the average permutation (black). C) Average circular-linear phase-MEP correlation for the five selected channels (blue) and the related permutation (black). Red dots indicate significance at alpha = 0.05 (cluster corrected). D) Phase-MEP relation extracting the instantaneous alpha phase via the Hilbert transform displaying the time course of the effect. Color coding identical to C). E) Phase correlation topography based on the Hilbert analysis. The strongest correlation was present at CP1. All shaded areas indicate the standard error of the mean. F) and G) are identical to D) and E) but for the beta frequency range (15–25 Hz).

Averaged Pearson linear correlations from EEG channels C3, FC1, FC5, CP1 and CP5 were performed between logarithmic power and both MEP amplitudes and permutated MEP amplitudes for frequencies between 2Hz and 30Hz in steps of 0.5Hz. We found a significant cluster (Fig 1A and 1B) including the frequency 10.4Hz to 16.7Hz (p = 0.028, cluster-statistics = 0.3624). The reported significant correlation was 0.0297 (r^2^ = 0.0009). When repeating the analyses for posterior channels we did not find a significant effect (no cluster found).

A-priori, we were interested in both alpha and beta effects, therefore we also performed an RM ANOVA directly testing for a possible interaction between alpha and beta power modulation. The RM ANOVA was a 2*2 ANOVA with the factors data (observed vs median permuted data) and frequency (alpha (8–12 Hz) and beta (15–25 Hz)). No interaction was found (F(1,26) = 1.45. p = 0.239). Since there was no interaction, we could not provide any evidence that the alpha power effect is be stronger than the beta power effect.

### Effect of neuronal oscillations phase on MEP amplitude

To investigate the relationship between neuronal oscillation phase and MEP amplitude, we compared correlations between phase of oscillation frequency and both measured and permuted MEP amplitude values for all frequencies between 2Hz and 30Hz. Correlations differed significantly for alpha frequencies ranging from 6.5Hz to 11.8Hz (*p* = 0.003). Statistics were corrected for multiple comparisons using cluster methods (Cluster-statistics = 0.1625; Fig 1C). The reported significant correlation was 0.1004 (r^2^ = 0.0101). No effects were found when repeating the analyses using the CSD transform (p > 0.05). When repeated for posterior channels we did not find a significant effect (p > 0.05).

Also for phase we performed a RM ANOVA analysis to investigate alpha-beta interactions. The interaction showed a trend (F(1,26) = 3.917, p = 0.059). This suggests that the effect of alpha is stronger, but that this cannot be fully corroborated with the statistic as it was borderline significant. Consistent with analyses in the main script, alpha showed a significant effect (t(1,26) = 3.57, p = 0.001), and beta did not show any effect (p>0.1).

### Inter-individual phase consistency related to high MEP amplitude

The phase consistency of the averaged individual dominant alpha frequency phase related to the 50% highest TMS-induced MEP amplitudes was analyzed for the five central EEG channels ipsilateral to the stimulation site. We did not find any phase consistency over participants (p > 0.1). We would like to note that using a different outlier criterion (standard deviations instead of a distance measure) did lead to significant phase consistency. However, as this was not consistent across outlier criteria we do not believe this to be a robust effect.

### Time course of alpha phase and power modulation

The time course extracted by the angle of the Hilbert transform of the filtered alpha data showed significant correlations between alpha phase and MEP amplitude (Fig 1D and 1E). Two clusters were identified (cluster 1: clusterstat = 0.887, -0.25–0 sec, p = 0.003; cluster 2: clusterstat = 0.673, -0.539 - -0.2773 sec, p = 0.018). The abrupt drop of phase correlation just before TMS onset is a consequence of edge effects of the filter (as the data was cut at zero). Still, it is evident that there is an increase in the correlation prior to the TMS pulse onset. We repeated the analyses for alpha power, we found one significant cluster (clusterstat = 2.185, -0.637 - -0.359 sec, p = 0.044). When repeating the analyses for posterior channels, we did not find a significant effect (p > 0.05 for both phase and power).

### Post-hoc analysis on time course of beta phase and power modulation

Besides using FFTs to estimate phase at stimulus onset, instantaneous phase has also often been estimated using the Hilbert transform. Therefore, as a post-hoc analyses, we analyzed the beta frequency window using the Hilbert transform to extract a time course around the beta phase modulation. For this analysis we found significant correlations between beta phase and MEP amplitude (Fig 1F and 1G). Four clusters were identified (cluster 1: clusterstat = 0.5045, -0.109–0 sec, p = 0.003; cluster 2: clusterstat = 0.488, -1.34 - -1.23 sec, p = 0.003; cluster 3: clusterstat = 0.361, -1.020 - -0.910 sec, p = 0.010; cluster 4: clusterstat = 0.291, -0.215 - -0.145 sec, p = 0.0270). We found no effect for beta power (all p > 0.1). To investigate the frequency specificity of the Hilbert phase analyses, we additionally ran the Hilbert analysis on filtered data at frequency ranges in which we did not expect any phase modulation (30–50 Hz, i.e. the theta band). No significant clusters were found (p > 0.05). When repeating the analyses for posterior channels we did not find a significant effect (p > 0.05 for both phase and power).

### Correction for pre-pulse EMG activity

**Fig 2:**
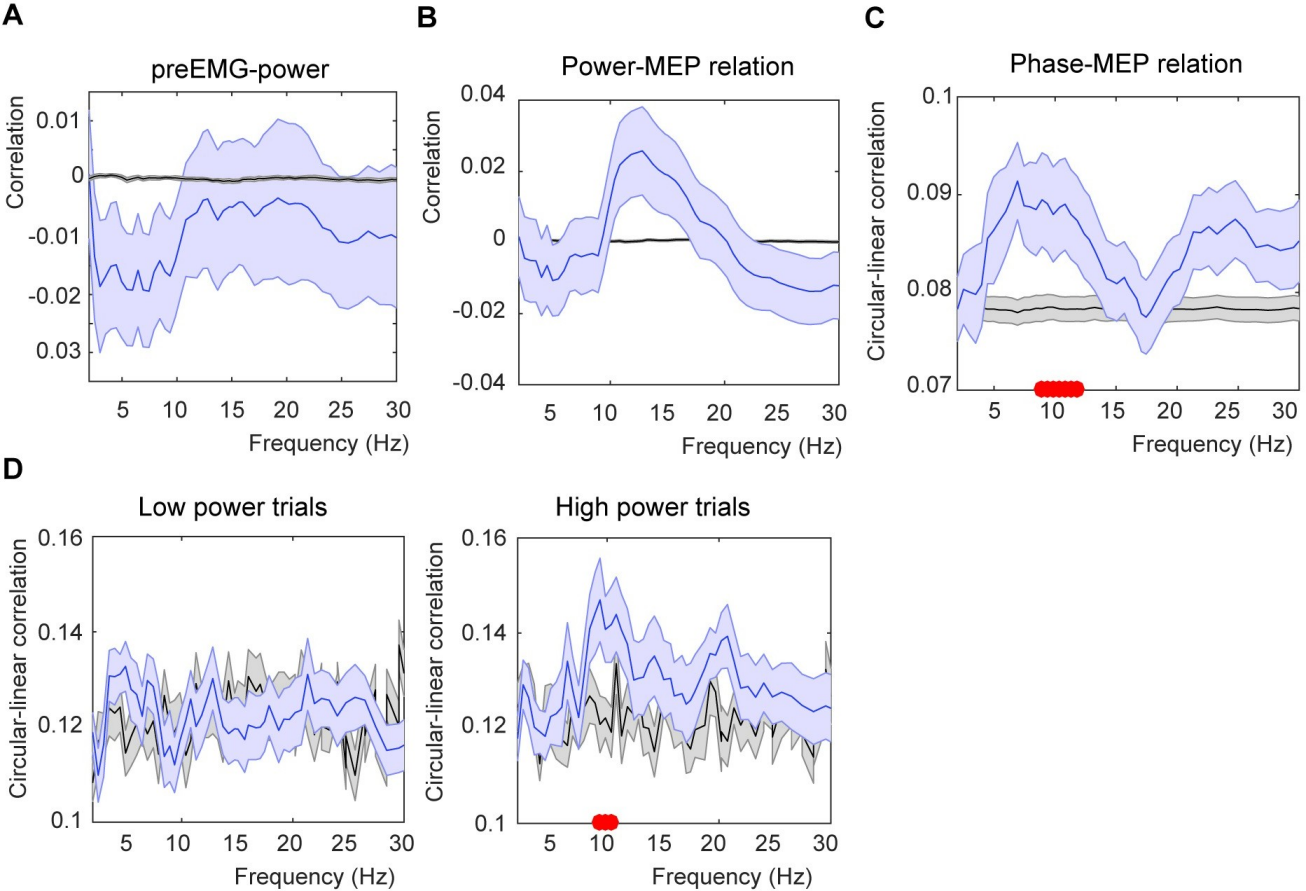
Analysis corrected for pre-TMS EMG activity and median split on power. A) Correlation between pre-TMS EMG activity and EEG power. B) Power-MEP size correlation using the residual power values (residuals of the correlation between power and pre-TMS EMG activity). C) Phase-MEP circular correlation using the residual MEP values (residuals of the correlation between pre-TMS EMG activity and MEP size). D) Phase-MEP circular correlation for either the low or high power trials. Conventions are the same as Fig 1.

The correlation between pre-pulse EMG activity and EEG showed a negative correlation for low frequencies (Fig 2A). This correlation did not survive correction for multiple comparison, but was significant uncorrected between 2.99 Hz (p = 0.036) and 9.37 Hz (p = 0.035). Note that not all datapoints within this frequency range were significant. When we corrected for the correlation, we only found small differences. For the power correlation, no cluster was found, but uncorrected p-values did show an effect, suggesting a trend (Fig 2B; uncorrected p-values of p = 0.044). For the phase-MEP relation, we found a significant cluster between 8.93 and 11.82 Hz (p = 0.032, clusterstatistics = 0.0711; Fig 2C).

### Phase correlation for high alpha power

Previous findings have shown stronger phase effects when power values are high [32]. To investigate if this was also true in our sample, we extracted the phase modulation again, but splitting the data for low and high power trials (median split for each frequency bin). We found a significant alpha phase modulation for the high (p = 0.03), but not the low power trials (p > 0.05). Comparing the high and low power effect directly (averaging over the significant high frequency cluster) revealed a significant difference between the high and low power trials (t(26) = 2.299, *p* = 0.030 (one-sided)). For the Hilbert transform, we repeated the same analyses averaging across the cluster closest to TMS pulse onset. Here, we did not find a significant difference between low and high power for either alpha or beta power (p > 0.1).

## Discussion

We tested whether corticospinal excitability is related to oscillations in the sensorimotor cortex. We report that the amplitude of MEPs induced by TMS at suprathreshold intensity (120% MT) is dependent on the instantaneous phase of ongoing alpha and beta frequency oscillations at the time and site of stimulation. Moreover, the alpha power also correlated with MEP amplitudes. In contrast to our expectations, we did not find any phase consistency linked to the 50% largest MEPs across participants.

While within participant there was a systematic relation between alpha phase and MEP, across participants we did not find any evidence of phase consistency. This finding is inconsistent with previous reports showing that the peak or trough of the signal should reflect the most excitable phase of the oscillation. Recent reports have emphasized the modulatory effect of μ-alpha phase on corticospinal excitability by demonstrating that larger MEPs are evoked during troughs compared with peaks of the μ-alpha waves [21, 22, 33, 34]. To address the question whether the phasic modulation of corticospinal excitability by μ-alpha reflects symmetric or asymmetric pulse facilitation or inhibition, Bergmann and colleagues [19] used real-time EEG-triggered single-pulse TMS to measure corticospinal excitability and paired-pulse TMS to assess short-latency intracortical inhibition (SICI). They found that MEP amplitudes were facilitated during μ-alpha troughs and rising slopes, but not during peaks and falling slopes. In addition, μ-alpha power and phase were not linked to intracortical inhibition. Therefore, sensorimotor alpha was related to pulsed facilitation, but not inhibition, of motor cortex excitability. Assuming that the trough of the oscillation wave measured at the scalp reflects the strongest local neuronal depolarization, facilitated MEP responses linked to the rising slope of sensorimotor alpha oscillations could reflect the responsiveness of the targeted neuronal ensembles to a synaptic input following rhythmic inhibition after the last neuronal population spike [35]. It is important to note here that the exact generators of EEG are unknown and consist of a summation of currents from many directions [36] and only direct in-vivo recordings of the local excitability can provide evidence for a link to depolarization of the underlying neuronal generators [37]. It is therefore possible that scalp measurements of phase are not consistent across participants (see e.g. [38] for a study which also shows on inconsistent phase estimations across participants in an EEG study). However, while we do not report phase consistency, previous studies stimulating at a specific phase do find effects [19, 39].

Based on our results and on previous findings, it appears that the sensorimotor alpha oscillation provides a cyclic modulation of excitability. Additionally, we found an effect of beta phase on MEP amplitudes. In a previous study from our lab, we found the rising slope phase relative to transcranial alternating current stimulation (tACS) in the beta frequency range to yield MEPs with the largest amplitudes [18]. This finding has been replicated by others [35]. It is interesting that the FFT analyses did not result in a significant beta phase effect, but the Hilbert analyses did. This could be a consequence of the general wider spread of individual beta frequency versus alpha peak [40] as the Hilbert analysis is robust against frequency variations.

We found a correlation between MEP amplitude and alpha power (Fig 1B). There have been conflicting results on whether alpha power and MEP size relate, some do report an effect [12, 13, 15–17] and other do not [41, 42]. These divergent findings reported in the literature show that a more mechanistic explanation about the exact origin of the measured μ/alpha generators is needed to understand the complex pattern that leads to spontaneous MEP modulations.

Several studies have investigated the relationship between neuronal oscillations and corticospinal excitability with varying methodological parameters, such as TMS intensity, TMS pulse waveform, inter pulse intervals, number of TMS pulses, targeted hand muscle, muscle contraction, channels for EEG recording or data analysis. One parameter that may be of particular interest for the discussion of the results presented here is TMS intensity. In contrast to the suprathreshold stimulation intensity (120% rMT) we applied, many previous studies applied TMS at individual MT intensity (100%; [12, 14, 15, 42], at low suprathreshold intensity (110% MT; [16, 17]) or at a pre-defined stimulation intensity that would lead to reliable MEPs of 1mV amplitude [13]. A systematical comparison of effects at different stimulation intensities found stronger effects for weaker intensities [22]. Note that in this study, the analysis focused on comparing peak versus trough stimulations, so it is possible that the optimal stimulation phase could be more consistent at lower intensities. We chose to apply TMS at suprathreshold intensities, as this ensures the induction of action potentials in the pyramidal neurons involved in the elicitation of the MEP and it allows for the assessment of facilitatory or inhibitory neuronal oscillation effects on successfully depolarized pyramidal neurons in form of increased or reduced MEP amplitude. Of course, with this approach a large neuronal extend of neighboring regions will also be stimulated this way, which can exhibit indirect effects on the MEP amplitude. In contrast, threshold stimulation limits the stimulation extend. At MT stimulation one can explore whether successful TMS induction of MEPs is dependent on ongoing neuronal oscillations. This is different to the investigation of the influence of neuronal oscillations on the actual magnitude of corticospinal excitability. One major disadvantage of investigating stimulation effects at MT is that many trials (by definition 50%) elicit no MEP. On these trials oscillatory modulations acting on cortical neurons are overlooked as only the downstream motor output is investigated. This can induce a non-linearity in the result that is not present in the cortex.

In our study we could only demonstrate a beta phase relationship when applying a Hilbert transform and not with the FFT approach. This adds to previous literature reporting differences in frequency relationships with phase modulation [16, 33, 42–45]. These differences could be explained by the strength of oscillatory power during TMS stimulation. In our study, TMS was applied during self-controlled muscle relaxation. Elevated levels of EEG beta activity over the motor and somatosensory cortex are usually linked to motor performance. However, the power of the ongoing resting EEG beta activity measured here is relatively low (Fig 1A), because participants are not performing any active motor task. As such, revealing any ongoing beta phase effect is challenging as phase is difficult to estimate, possibly leading to false negatives. Indeed, in a study by Torrecillos and colleagues [44], MEP size was modulated by beta phase when restricting TMS triggering to trials with high beta power. This is similar to other online-triggered TMS studies in which TMS is often only applied when power reaches a certain threshold [19, 33]. tACS does induce phase modulations at beta ranges [18, 39, 46], likely because those oscillatory beta patterns are induced by the stimulation itself. Other EEG studies do report beta-phase effects when using different TMS stimulation parameters, such as threshold stimulation [16, 42]. For future studies, it is critical to verify the exact mechanisms behind this variation: Is the absence of phase modulation during low power a consequence of the inability to reliably estimate oscillatory phase or a physiological change in how phase modulates neuronal responses? Mechanistic explanations of varying TMS parameters are lacking, as well as a good control on how the state of the circuitry might have a direct effect on the reported frequency ranges.

Previous work on the variability of MEP responses have also focused on the level of contraction of relevant muscle groups at the time of TMS stimulation [47]. This literature reported more variability at lower muscle contraction levels [47–49] and overall stronger MEPs at higher muscle pre-contraction [50]. Here, we showed that the contraction variations did not vary consistently in the frequency ranges of the significant effects. Moreover, correcting between EEG power and pre-pulse EMG activity did also not change our effects. In other words, any pre-pulse EMG activity that is directly related to EEG power cannot explain our effect.

In the current study, the individually dominant frequencies were measured over sensorimotor areas. The location of the alpha effect was directly underlying the stimulation site, which prompts us to cautiously speculate that we are indeed extracting the influence of underlying excitability changes on the μ-rhythms known to be critical for motor processing [51]. The main results presented here concern a common reference approach in which data is re-referenced to the average of all EEG channels. The oscillatory activity measured in this way is more sensitive to global activity patterns and less sensitive to local patterns. These patterns are more specifically picked up by spatial transforms such as the CSD transforms. Indeed, a common way to online-triggered TMS on the motor system is to use such a Hjorth or CSD transformation [19, 22, 33]. It is therefore puzzling that we do not find a phase modulation in our FFT analyses when applying a CSD transformation. However, we did not find alpha phase effects for more posterior and occipital channels, which are the core generators of posterior alpha. It is therefore unlikely that posterior alpha is driving the effect. The discrepancy between our results and previous reports maybe due to the specifics of the stimulation (stimulation at higher alpha power values for the online-triggered stimulation). Although higher local specificity of the oscillatory activity might seem as a benefit, the downside of this transformation is that any activity patterns shared over a larger brain area could be dampened, missing the more global effects as we report here.

## Conclusion

We show that MEP amplitude is dependent on the phase of dominant sensorimotor alpha and beta frequency oscillations and alpha power at the time and site of TMS. While our results support the notion for a relationship between particular neuronal oscillation patterns and TMS induced MEPs, more research is needed for an accurate understanding of how frequency phase and power are related to cortical excitability. This is crucial considering that the reported correlations are very low and therefore not clinically relevant at this stage. Eventually, we will be able to incorporate these neurophysiological measures into TMS protocols for more specific applications and more dependable outcome measures. Advanced knowledge about the functional mechanisms of cortical and corticospinal excitability will help to develop efficient closed-loop protocols combining online neurophysiological measures with TMS [33, 52]. Ultimately, such information based TMS protocols will have great potential to provide reliable and well controlled results for research purposes and they could be further developed to serve as specified monitoring and diagnostic tools or allow for individualized treatment applications in clinical settings.

## Supporting information

S1 Dataset (ZIP)
