# Supplementary material for: Phase and power modulations on the amplitude of TMS-induced motor evoked potentials
Source: PLoS One. 2021 Sep 16;16(9):e0255815. doi: 10.1371/journal.pone.0255815 (PMC8445484; doi:10.1371/journal.pone.0255815)
Supplement: Peer review history

# Peer review: Decision Letter 0

**DOI:** 10.1371/journal.pone.0255815.r001

**Authors:** Luigi Cattaneo

7 Apr 2021

PONE-D-21-07587

Oscillatory phase modulates the amplitude of TMS-induced motor evoked potentials

PLOS ONE

Dear Dr. Ten Oever,

Thank you for submitting your manuscript to PLOS ONE. After careful consideration, we feel that it has merit but does not fully meet PLOS ONE’s publication criteria as it currently stands. Therefore, we invite you to submit a revised version of the manuscript that addresses the points raised during the review process.

Please submit your revised manuscript by May 22 2021 11:59PM. If you will need more time than this to complete your revisions, please reply to this message or contact the journal office at plosone@plos.org. When you're ready to submit your revision, log on to https://www.editorialmanager.com/pone/ and select the 'Submissions Needing Revision' folder to locate your manuscript file.

Please include the following items when submitting your revised manuscript:

- A rebuttal letter that responds to each point raised by the academic editor and reviewer(s). You should upload this letter as a separate file labeled 'Response to Reviewers'.
- A marked-up copy of your manuscript that highlights changes made to the original version. You should upload this as a separate file labeled 'Revised Manuscript with Track Changes'.
- An unmarked version of your revised paper without tracked changes. You should upload this as a separate file labeled 'Manuscript'.

If you would like to make changes to your financial disclosure, please include your updated statement in your cover letter. Guidelines for resubmitting your figure files are available below the reviewer comments at the end of this letter.

If applicable, we recommend that you deposit your laboratory protocols in protocols.io to enhance the reproducibility of your results. Protocols.io assigns your protocol its own identifier (DOI) so that it can be cited independently in the future. For instructions see: http://journals.plos.org/plosone/s/submission-guidelines#loc-laboratory-protocols. Additionally, PLOS ONE offers an option for publishing peer-reviewed Lab Protocol articles, which describe protocols hosted on protocols.io. Read more information on sharing protocols at https://plos.org/protocols?utm_medium=editorial-email&utm_source=authorletters&utm_campaign=protocols.

We look forward to receiving your revised manuscript.

Kind regards,

Luigi Cattaneo, MD, PhD

Academic Editor

PLOS ONE

Journal Requirements:

When submitting your revision, we need you to address these additional requirements.

1. Please ensure that your manuscript meets PLOS ONE's style requirements, including those for file naming. The PLOS ONE style templates can be found at

https://journals.plos.org/plosone/s/file?id=wjVg/PLOSOne_formatting_sample_main_body.pdf and

https://journals.plos.org/plosone/s/file?id=ba62/PLOSOne_formatting_sample_title_authors_affiliations.pdf

2.  We note that you have indicated that data from this study are available upon request. PLOS only allows data to be available upon request if there are legal or ethical restrictions on sharing data publicly. For more information on unacceptable data access restrictions, please see http://journals.plos.org/plosone/s/data-availability#loc-unacceptable-data-access-restrictions.

In your revised cover letter, please address the following prompts:

a) If there are ethical or legal restrictions on sharing a de-identified data set, please explain them in detail (e.g., data contain potentially sensitive information, data are owned by a third-party organization, etc.) and who has imposed them (e.g., an ethics committee). Please also provide contact information for a data access committee, ethics committee, or other institutional body to which data requests may be sent.

b) If there are no restrictions, please upload the minimal anonymized data set necessary to replicate your study findings as either Supporting Information files or to a stable, public repository and provide us with the relevant URLs, DOIs, or accession numbers. For a list of acceptable repositories, please see http://journals.plos.org/plosone/s/data-availability#loc-recommended-repositories.

We will update your Data Availability statement on your behalf to reflect the information you provide.

[Note: HTML markup is below. Please do not edit.]

Reviewers' comments:

Reviewer's Responses to Questions

**Comments to the Author**

1. Is the manuscript technically sound, and do the data support the conclusions?

The manuscript must describe a technically sound piece of scientific research with data that supports the conclusions. Experiments must have been conducted rigorously, with appropriate controls, replication, and sample sizes. The conclusions must be drawn appropriately based on the data presented. 

Reviewer #1: Partly

Reviewer #2: Yes

**********

2. Has the statistical analysis been performed appropriately and rigorously? 

Reviewer #1: N/A

Reviewer #2: Yes

**********

3. Have the authors made all data underlying the findings in their manuscript fully available?

The PLOS Data policy requires authors to make all data underlying the findings described in their manuscript fully available without restriction, with rare exception (please refer to the Data Availability Statement in the manuscript PDF file). The data should be provided as part of the manuscript or its supporting information, or deposited to a public repository. For example, in addition to summary statistics, the data points behind means, medians and variance measures should be available. If there are restrictions on publicly sharing data—e.g. participant privacy or use of data from a third party—those must be specified.

Reviewer #1: Yes

Reviewer #2: No

**********

4. Is the manuscript presented in an intelligible fashion and written in standard English?

PLOS ONE does not copyedit accepted manuscripts, so the language in submitted articles must be clear, correct, and unambiguous. Any typographical or grammatical errors should be corrected at revision, so please note any specific errors here.

Reviewer #1: Yes

Reviewer #2: Yes

**********

5. Review Comments to the Author

Please use the space provided to explain your answers to the questions above. You may also include additional comments for the author, including concerns about dual publication, research ethics, or publication ethics. (Please upload your review as an attachment if it exceeds 20,000 characters)

Reviewer #1: The authors performed an experiment with the aim to associate power and phase of 2-30 Hz frequencies of the EEG recordings, in a pre-TMS window, with the amplitude of the evoked-TMS MEPs. They found that a significant correlation exists only between phase and MEP amplitude at alpha and beta frequencies. This correlation survives the exclusion of pre-TMS EMG background contraction, They aimed to show that a specific range of phases (correlating with MEP amplitude), was consistent across the participants, and eventually that phase and power in the alpha range interact, with only high power alpha showing significant correlation with MEPs amplitude.

While this is an interesting paper and topic, the manuscript presents more than one methodological concern and I would appreciate if the authors clarify some choices

1) It is not clear what the task of the participants was. Moreover, it would be more transparent (and in general easier for the reader) if the authors (briefly) described the experiment within which the sessions described in the present manuscript were recorded (other than just pointing the reader to the references).

2) line 114 pg 6: I believe that relying on SD to exclude trials is a sub-optimal approach. From Jones 2019, "sample mean and variance are easily distorted by extreme values, meaning that more distant outliers may ‘mask’ lesser ones" (Jones 2019, https://doi.org/10.3758/s13414-019-01726-3). Other more robust measures, such as "Sn", that can be applied also to skewed distributions (see the reference which offers also a MATLAB function for this purpose).

Moreover, is there a reason for excluding the lowest pre-TMS contractions, assuming that the authors are looking for neurophysiological relationships while participants are at rest?

2a) line 119: again, assuming that the authors are looking for a relationship while participants are at rest, it is reasonable that trials showing extreme pre-TMS contractions were excluded. However, I do not see the reason of excluding high amplitude MEPs. I understand removing trials in which no MEP is detected, because it might add noise to the data (maybe in that trial the coil has been moved from the hot spot, maybe it was not properly tilted and tangentially held for example), not knowing if the peak-to-peak measures are actually come from very small MEPs or not. Ideally the best solution would be to use a more robust correlation metric such as the spearman correlation without excluding data (at least for the power analyses). I would like the authors to explain the rationale behind their choice.

3) line 130 , pg 7: it is not entirely clear to me why the logarithm of the power has been chosen as a measure of EEG frequency amplitude. One could have chosen the amplitude, the power, or other transformations. This choice implies that the relationship between log(power EEG freqs) vs MEP amplitude is linear. Elaborate on this point please.

Again, if no linear relationship is reasonably assumed, a spearman correlation should be considered: indeed, given the assumed monotonic relationship between EEG power and MEP amplitudes, should provide a positive/negative correlation even if the the relationship is not linear.

4) line 145 pg 8: the authors have chosen to average 5 channels around C3. This should be consistent throughout the analyses: either they chose the average of the channels or each channel should be analysed separately (or they can be taken into consideration in a cluster based statistics which includes also the position of the channels, see fieldtrip website). Specifically I am referring to line 159 about the consistency across participants, where the channels have been treated separately and corrected by Bonferroni. Elaborate on this issue please if there is a rationale behind this choice, otherwise adopt only one criterion.

5) In general however, given the EEG volume conduction problems, there is no sufficient evidence in my opinion that effects found arise from central sources. Since the authors have chosen to average 5 "central" electrodes, they should compare the effects not only against a permutation, but also against other 5 occipito-parietal sensors on the same side of the stimulation. Indeed, as acknowledged by authors, a prominent alpha rhythm is present also in more posterior brain sites. Even for Beta analyses, such a comparison would provide a better evidence that the correlation effects do not arise caused by occipito-parietal.

6) line 147: why was the median used for the permuted data and the average for the actual data?

7) line 150 pg 8: this is just a check of whether the authors have used the error 1 threshold correctly, given that fieldtrip might be misleading concerning this issue:

https://www.fieldtriptoolbox.org/faq/why_should_i_use_the_cfg.correcttail_option_when_using_statistics_montecarlo/

(the authors might specify in the manuscript the cfg.correcttail used, so that the solution adopted is more transparent)

8) line 189 pg 9: the authors state that CP1 has the strongest correlation. Is this comparison tested statistically?

9) line 196: again, I do not see the reason for treating the channels separately when they have been averaged for the previous power/phase-MEP analyses.

10) line 323: beta power is relatively low with respect to what? in this case the hilbert should be performed again separately for high and low power data? (the alpha as well, given the obtained results between power and phase)

11) can the authors interpret the time-course of the correlation between the hilbert of the beta phases and MEP amplitude? for example, a significant correlation has been found more than one second before TMS pulse; is it something the authors expected? Also the sudden jumping of the correlations looks peculiar, especially considering the very short significant period just before the TMS pulse (in contrast to the alpha hilbert, which shows a longer period of correlation before the TMS).

12) Figure 1A: it is not clear what the inset is, please explain more in detail. Moreover, the inset cannot be apporciated given the resolution of the figure (see comment 13)

13) Figure 1B: it looks like the image is lacking the error bar for the permuted data (grey). (the image on the PDF is very blurry, likely not authors' fault)

14) Please in figure 1 F and H indicate the 5 target sensors position

Reviewer #2: In their manuscript (MS), Schilberg and colleagues employ single pulse Transcranial Magnetic Stimulation (TMS) over the primary motor cortex (M1), together with simultaneous Electroencephalography (EEG) and Electromyography (EMG) to show that the amplitude of motor evoked potentials (MEPs) correlates with the phase of the ongoing EEG activity in the alpha and beta frequency band.

The present MS is not very novel per se, as the state-dependency of MEPs and TEPs (but also the occurrence of phosphines when stimulation V1 see for example Romei J Neurosc 2010) is a topic of debate since early nineties (Kiers Clin Neurophys 1993) and has been tackled by many different authors in the last 30 years (some of them cited in the MS, ref from 8 to 23). Nevertheless, this study is methodologically solid and it reports few new results that might be useful for the scientific community.

I have just few major and minor concerns that, in my opinion, need to be addressed in order to guarantee a better interpretation of the results. I have detailed my comments below:

MAJORS

1) The authors focus on alpha and beta rhythms because “alpha and beta frequency range have been linked to sensorimotor processing (8-11)”. They also report previous works showing “…diverse associations of corticospinal excitability with preceding oscillation frequency power and phase…. Reported findings include both the existence and absence of relationships between MEP amplitude and alpha or beta frequency power (12-18), phase (19-22) and phase-power interaction (23)”. Many of the reported works refer to mu-band that, however, is by definition “multispectral” (Tihonen 1998). This means that it includes frequencies in the alpha (8-12Hz) and beta (12.5-30Hz), which may overlap and interact. In line with this, the authors correctly analysed separately alpha and beta. I think that it would be interesting to see whether there is any statistical interactions between alpha and beta, both in power and in phase (e.g. multivariate regression analysis?).

2) When analyzing the inter-individual phase consistency related to high MEP amplitude the authors found significant results only in CP1 and not in the other considered channels (after corrections). Even if the number of EEG contact is limited, perhaps, moving from the voltage space to the source space (MNI could be ok, if the MRI of single subjects are not available) could lead to more solid results.

3) When considering the inter-individual phase consistency related to high MEP amplitude the authors focused on the 50% amplitude of the MEP. Please justify this a priori selection.

4) In Figure 1F and H the topographies are not very clear. Perhaps I would saturate the colour-scale and/or I would set to zero the non-significant values.

5) - This is optional - Fecchio et al. (Plos One, 2017) showed that the EEG responses to TMS (TEPs) are different in presence/absence of MEP. Since the authors collect the EEG during TMS, It would very be interesting to test whether not only the MEPs, but also the TEPs are modulated by the phase of the ongoing activity.

MINORS

1) LINE 104 ”The EEG and EMG data analyzed for this study is part of a larger TMS study that has been analyzedand published separately (24). The data was collected during a single control session of that largerstudy, which included one block of repetitive sham TMS, but no other form of real TMS in addition to the experimental single TMS pulses included in the analysis.” This sentence is reported in the analysis section, I would report that in the procedure. I would also clearly indicate a the beginning of the Methods that these data have been collected in the context of a previous study.

2) Please report the protocol number for the Ethical approval and indicate whether (I hope so) the participants signed any informed consent.

**********

6. PLOS authors have the option to publish the peer review history of their article (what does this mean?). If published, this will include your full peer review and any attached files.

If you choose “no”, your identity will remain anonymous but your review may still be made public.

**Do you want your identity to be public for this peer review?** For information about this choice, including consent withdrawal, please see our Privacy Policy.

Reviewer #1: No

Reviewer #2: No

[NOTE: If reviewer comments were submitted as an attachment file, they will be attached to this email and accessible via the submission site. Please log into your account, locate the manuscript record, and check for the action link "View Attachments". If this link does not appear, there are no attachment files.]

While revising your submission, please upload your figure files to the Preflight Analysis and Conversion Engine (PACE) digital diagnostic tool, https://pacev2.apexcovantage.com/. PACE helps ensure that figures meet PLOS requirements. To use PACE, you must first register as a user. Registration is free. Then, login and navigate to the UPLOAD tab, where you will find detailed instructions on how to use the tool. If you encounter any issues or have any questions when using PACE, please email PLOS at figures@plos.org. Please note that Supporting Information files do not need this step.

# Author comment: Author response to Decision Letter 0

**DOI:** 10.1371/journal.pone.0255815.r002

27 May 2021

We thank the reviewers for their useful comments. We have answered all concerns point by point below. Changes in the manuscript have been highlighed in yellow.

Reviewer #1: The authors performed an experiment with the aim to associate power and phase of 2-30 Hz frequencies of the EEG recordings, in a pre-TMS window, with the amplitude of the evoked-TMS MEPs. They found that a significant correlation exists only between phase and MEP amplitude at alpha and beta frequencies. This correlation survives the exclusion of pre-TMS EMG background contraction, They aimed to show that a specific range of phases (correlating with MEP amplitude), was consistent across the participants, and eventually that phase and power in the alpha range interact, with only high power alpha showing significant correlation with MEPs amplitude.

While this is an interesting paper and topic, the manuscript presents more than one methodological concern and I would appreciate if the authors clarify some choices

1) It is not clear what the task of the participants was. Moreover, it would be more transparent (and in general easier for the reader) if the authors (briefly) described the experiment within which the sessions described in the present manuscript were recorded (other than just pointing the reader to the references).

We thank the reviewer for this valuable suggestion which we gladly implanted in the revised version of the manuscript. We now describe the experiment in which this data was recorded in more detail in order to provide the broader context of the experimental session here analyzed. We also explicitly stated now that measurements were done with participants being at rest and thus without the execution of a specific task. Based on a comment by another reviewer, we moved this paragraph to the beginning of the Methods section to further increase clarity of this general aspect of study design and experimental context. 

2) line 114 pg 6: I believe that relying on SD to exclude trials is a sub-optimal approach. From Jones 2019, "sample mean and variance are easily distorted by extreme values, meaning that more distant outliers may ‘mask’ lesser ones" (Jones 2019, https://doi.org/10.3758/s13414-019-01726-3). Other more robust measures, such as "Sn", that can be applied also to skewed distributions (see the reference which offers also a MATLAB function for this purpose).

Moreover, is there a reason for excluding the lowest pre-TMS contractions, assuming that the authors are looking for neurophysiological relationships while participants are at rest?

2a) line 119: again, assuming that the authors are looking for a relationship while participants are at rest, it is reasonable that trials showing extreme pre-TMS contractions were excluded. However, I do not see the reason of excluding high amplitude MEPs. I understand removing trials in which no MEP is detected, because it might add noise to the data (maybe in that trial the coil has been moved from the hot spot, maybe it was not properly tilted and tangentially held for example), not knowing if the peak-to-peak measures are actually come from very small MEPs or not. Ideally the best solution would be to use a more robust correlation metric such as the spearman correlation without excluding data (at least for the power analyses). I would like the authors to explain the rationale behind their choice.

We believe that in general a proper outlier detection is important to ensure robust estimation of any effect of interest. If we do not remove statistical outliers according to standard and accepted outlier analysis criteria, our findings are likely inappropriately affected by individual values. We therefore originally decided to conduct an outlier analysis following standard statistical criteria, excluding outlier values for both high power as well as MEPs since both measurements are used in the calculated correlation. 

Regarding the outlier criteria for pre-TMS contractions, we indeed excluded trials with pre-TMS contractions that were 3.5 times higher than the standard deviation from the average of all pre-pulse EMG peak-to-peak values for a time window of 100ms prior to TMS. There were no exclusions of trials based on low pre-TMS contractions. However, we did exclude trials with MEP amplitudes lower than 0.05mV, as those do not represent an elicited MEP. We clarified this in the manuscript. 

Indeed, there might be different ways to deal with outlier criteria. One thing the reviewer suggests instead of using the standard deviation, to rather rely on Spearman correlation without outlier removal. While this is possible for the power-MEP correlation, we are not aware of a circular correlation that can also perform non-parametric correlation measures such as the Spearman correlation. The only option would be to use permutations to estimate the circular correlation as expected by chance. But this is already the approach we had taken in the current manuscript. (We calculate the correlation both for the observed data as well as permuted values of this approach and then use cluster statistics to investigate whether the chance permutation is higher than the observed correlation).

To still ensure that none of our results are due to specific outlier criteria, we re-run the analysis in two different ways (as suggested by the reviewer):

- Perform no outlier correction for high power or high MEP and perform a Spearman correlation (for the phase correlation still a circular correlation was used). 

- Perform the outlier correction with an Sn approach. 

Overall, our general results remained, with some notable additions and differences we now also report in the revised manuscript. Please see the table below. 

For the FFT alpha power, the previously trend-significant effect reached significance in the Sn outlier approach (p = 0.028). This also was the case for the alpha Hilbert analyses in which previously the cluster was not significant, but now reached significance (p=0.04). 

 Original; No outliers removal + Spearman; Sn outlier approach

FFT phase: p = 0.028; p = 0.029; p = 0.003

FFT power: No cluster found; No cluster found; p = 0.028

Hilbert alpha phase: p = 0.002; p = 0.003; p = 0.003

Hilbert alpha power: p > 0.1; p > 0.05; p = 0.04

Hilbert beta phase p < 0.001 p = 0.002 p = 0.003

Hilbert beta power p > 0.1 p > 0.1 P > 0.1

As the reviewer pointed out, the Sn outlier approach might be the most appropriate for outlier detection. Therefore, we now report all results using this approach. Besides the change of the alpha power effect, we also saw a change in the consistency of the phase estimation across subjects. Both for the “no outlier removal” and “Sn outlier removal” approach this effect did not reach significance. We therefore decided to report this transparently stating that this effect depends on the outlier criteria chosen, but further do not dedicate a figure to it.

Changes in the manuscript are: 

- The title changed and now reads: “Phase and power modulations on the amplitude of TMS-induced motor evoked potentials”.

- All results reflecting the phase consistency have been removed. The results section regarding the manuscript now reads: “The phase consistency of the averaged individual dominant alpha frequency phase related to the 50% highest TMS-induced MEP amplitudes was analyzed for the five central EEG channels ipsilateral to the stimulation site. We did not find any phase consistency over participants (p > 0.1). We would like to note that using a different outlier criterion (standard deviations instead of a distance measure) did lead to significant phase consistency. However, as this was not consistent across outlier criteria we do not believe this to be a robust effect. “

- We now report on the significant alpha power effect in the discussion and the abstract. 

3) line 130 , pg 7: it is not entirely clear to me why the logarithm of the power has been chosen as a measure of EEG frequency amplitude. One could have chosen the amplitude, the power, or other transformations. This choice implies that the relationship between log(power EEG freqs) vs MEP amplitude is linear. Elaborate on this point please.

Again, if no linear relationship is reasonably assumed, a spearman correlation should be considered: indeed, given the assumed monotonic relationship between EEG power and MEP amplitudes, should provide a positive/negative correlation even if the the relationship is not linear.

The log transform is the most commonly accepted EEG power transform to ensure transform of the skewed data to a more normal distribution (see e.g. (Gasser, Bächer, & Möcks, 1982; Smulders, Ten Oever, Donkers, Quaedflieg, & van de Ven, 2018)). We did show a significant effect based on the suggested Sn outlier approach, which was absent (or uncorrected trend-significant) using the Spearman correlation. This suggest that the relation is indeed linear between the log distribution of the alpha power and the MEP power. 

4) line 145 pg 8: the authors have chosen to average 5 channels around C3. This should be consistent throughout the analyses: either they chose the average of the channels or each channel should be analysed separately (or they can be taken into consideration in a cluster based statistics which includes also the position of the channels, see fieldtrip website). Specifically I am referring to line 159 about the consistency across participants, where the channels have been treated separately and corrected by Bonferroni. Elaborate on this issue please if there is a rationale behind this choice, otherwise adopt only one criterion.

We agree with the reviewer. We first went and redid the analyses across all five channels. The phase consistency was significant and consistent (Z=3.37, p=0.03). However, as we report in comment to point #2, the phase consistency did not survive the application of a different outlier criterion and we were therefore decided not to conclusively report on this effect. We mention that we don’t find strong evidence for phase consistency in the results section. 

5) In general however, given the EEG volume conduction problems, there is no sufficient evidence in my opinion that effects found arise from central sources. Since the authors have chosen to average 5 "central" electrodes, they should compare the effects not only against a permutation, but also against other 5 occipito-parietal sensors on the same side of the stimulation. Indeed, as acknowledged by authors, a prominent alpha rhythm is present also in more posterior brain sites. Even for Beta analyses, such a comparison would provide a better evidence that the correlation effects do not arise caused by occipito-parietal.

We have repeated the analyses for 5 occipito-parietal sensors (O1, P3, P7, Pz, T7). For the FFT analyses we found no effect (for phase p > 0.05; for power no cluster was found). For the alpha Hilbert we also did not find any significant effect for phase or power in alpha or beta (cluster p-value > 0.05). We have included this analysis in the main manuscript. All results have been updated to add this information. In addition, we report in the discussion on this finding by stating: “...we did not find alpha phase effects for more posterior and occipital channels, which are the core generators of posterior alpha. It is therefore unlikely that posterior alpha is driving the effect.”

6) line 147: why was the median used for the permuted data and the average for the actual data?

The mean refers to the mean of the five channels. This was performed for both the actual and the permuted data. The median refers to the median of the 1000 permutations we performed which we use to contrast the actual data to. For each of these permutations the mean of the five channels was still used. We clarified this now in the manuscript. It now reads: “Correlation values at each of these channels were averaged for the initial analysis. We created a null distribution repeating the analysis using permuted labels. This null distribution reflects the expected average correlation based on chance. To statistically compare our observed values with the chance values, we compared the median of the permuted labels with the observed correlation values for power and phase separately at all frequency bins of 0.5Hz between 2Hz and 30Hz”.

7) line 150 pg 8: this is just a check of whether the authors have used the error 1 threshold correctly, given that fieldtrip might be misleading concerning this issue:

https://www.fieldtriptoolbox.org/faq/why_should_i_use_the_cfg.correcttail_option_when_using_statistics_montecarlo/

(the authors might specify in the manuscript the cfg.correcttail used, so that the solution adopted is more transparent)

We regret not being fully transparent and for this oversight. Indeed, fieldtrip by default corrects the alpha of the values and not the probabilities and we used this default. While for the phase correlations this does not change anything (as these correlations can only be positive so a-priori only positive clusters are expected), for the power correlations this does have an influence. For consistency across the phase and power effects, we now report all the corrected p-values which changes the power p-values throughout the manuscript. We also state this in the manuscript. It reads: “For the phase analysis we report the one-sided p-value as circular correlations can only be positive. For the power analysis we report the corrected p-values.” We thank the reviewer for this attentive comment. 

8) line 189 pg 9: the authors state that CP1 has the strongest correlation. Is this comparison tested statistically?

Good point. We did not test this statistically and thus removed this description from the text.

9) line 196: again, I do not see the reason for treating the channels separately when they have been averaged for the previous power/phase-MEP analyses.

We agree and changed this accordingly.

10) line 323: beta power is relatively low with respect to what? in this case the hilbert should be performed again separately for high and low power data? (the alpha as well, given the obtained results between power and phase)

The beta power was low in our study in comparison with other studies in which participants were actively required to perform a motor task. This is also demonstrated by the FFT that does not show a clear beta peak (see Fig. 1A). To make it clearer that the EEG beta power measured here is rather low, because there was no motor task involved, we updated the text to: “In our study, TMS was applied during self-controlled muscle relaxation. Elevated levels of EEG beta activity over the motor and somatosensory cortex are usually linked to motor performance. However, the power of the ongoing resting EEG beta activity measured here is relatively low (Fig. 1A), because participants are not performing any active motor task.” 

As the reviewer suggested, we repeated the analyses for low and high alpha/beta power for the Hilbert analyses (averaged of the cluster closed to TMS pulse onset). We did not find a significant difference between low and high power trials (p>0.1). This is now reported in the manuscript.

11) can the authors interpret the time-course of the correlation between the hilbert of the beta phases and MEP amplitude? for example, a significant correlation has been found more than one second before TMS pulse; is it something the authors expected? Also the sudden jumping of the correlations looks peculiar, especially considering the very short significant period just before the TMS pulse (in contrast to the alpha hilbert, which shows a longer period of correlation before the TMS).

We do not have a clear interpretation for this effect. As indicated in the manuscript the beta Hilbert analyses was a post-hoc analyses we only performed after we also performed the alpha Hilbert analyses. Our a-priori analyses focused on the FFT. But after some review rounds we also decided to post-hoc include the beta Hilbert transform, which in contrast to the FFT analyses does show an effect. We agree that the finding in the beta is rather peculiar and difficult to explain. It is possible that this is related to some form of cross-frequency coupling by which the beta power is coupled to lower frequency phase. This has been reported in the literature (Axmacher et al., 2010; Canolty et al., 2006). Unfortunately, at this moment this is rather speculative and we do not have a satisfying answer to why this is the case. 

12) Figure 1A: it is not clear what the inset is, please explain more in detail. Moreover, the inset cannot be apporciated given the resolution of the figure (see comment 13)

The inset refers to the ERPs related to different phase bins to provide a visualization of the different alpha phases we estimated ensuring that the phases are not biased. We have changed the figure legend to make this clearer.

13) Figure 1B: it looks like the image is lacking the error bar for the permuted data (grey). (the image on the PDF is very blurry, likely not authors' fault)

We ensure that the error bars are there. However, they are very similar across participants (correlations by chance are close to zero, and circular-linear correlations by chance are very similar when trial amounts are comparable).

14) Please in figure 1 F and H indicate the 5 target sensors position

We have added the sensor positions to the figure.

 

Reviewer #2: In their manuscript (MS), Schilberg and colleagues employ single pulse Transcranial Magnetic Stimulation (TMS) over the primary motor cortex (M1), together with simultaneous Electroencephalography (EEG) and Electromyography (EMG) to show that the amplitude of motor evoked potentials (MEPs) correlates with the phase of the ongoing EEG activity in the alpha and beta frequency band.

The present MS is not very novel per se, as the state-dependency of MEPs and TEPs (but also the occurrence of phosphines when stimulation V1 see for example Romei J Neurosc 2010) is a topic of debate since early nineties (Kiers Clin Neurophys 1993) and has been tackled by many different authors in the last 30 years (some of them cited in the MS, ref from 8 to 23). Nevertheless, this study is methodologically solid and it reports few new results that might be useful for the scientific community.

I have just few major and minor concerns that, in my opinion, need to be addressed in order to guarantee a better interpretation of the results. I have detailed my comments below:

MAJORS

1) The authors focus on alpha and beta rhythms because “alpha and beta frequency range have been linked to sensorimotor processing (8-11)”. They also report previous works showing “…diverse associations of corticospinal excitability with preceding oscillation frequency power and phase…. Reported findings include both the existence and absence of relationships between MEP amplitude and alpha or beta frequency power (12-18), phase (19-22) and phase-power interaction (23)”. Many of the reported works refer to mu-band that, however, is by definition “multispectral” (Tihonen 1998). This means that it includes frequencies in the alpha (8-12Hz) and beta (12.5-30Hz), which may overlap and interact. In line with this, the authors correctly analysed separately alpha and beta. I think that it would be interesting to see whether there is any statistical interactions between alpha and beta, both in power and in phase (e.g. multivariate regression analysis?).

Thank you for this excellent suggestion. Indeed, we extracted alpha (8-12 Hz) and beta (15-25 Hz) phase correlation from the FFT and performed a repeated measures anova with factors frequency (alpha versus beta) and permutation (original data versus permutation data). The interaction showed a trend (F(1,26) = 3.917 = 0.059). This suggests that the effect of alpha is stronger, but that this cannot be fully corroborated with the statistic. Consistent with analyses in the main script, alpha showed a significant effect (t(1,26) = 3.57, p = 0.001), and beta did not show any effect (p>0.1). For power we did not find an interaction (F(1,26)=1.45, p =0.239). We report this analysis in the updated manuscript.

The absence of an interaction is not strange considering that beta phase likely does have an influence on the MEP size. In our FFT analyses this did not come out, but for the Hilbert analysis, the phase correlation in beta did show an effect. We also repeated the interaction analysis for the Hilbert transform across time, but did not find any effects here.

2) When analyzing the inter-individual phase consistency related to high MEP amplitude the authors found significant results only in CP1 and not in the other considered channels (after corrections). Even if the number of EEG contact is limited, perhaps, moving from the voltage space to the source space (MNI could be ok, if the MRI of single subjects are not available) could lead to more solid results.

In our response to a comment from reviewer 1 with regard to the outlier analysis criteria, we repeated the analyses using an alternative outlier criterium which revealed that our finding of phase consistency measure was not as robust as the previous statistics seemed to suggest. While the effect seemed quite strong, especially on CP1 (even correcting for multiple comparisons using Bonferonni), using a different outlier criterion removed the effect completely. This surprised us and we decided the best way forward is to report on this.

The manuscript now reads: “The phase consistency of the averaged individual dominant alpha frequency phase related to the 50% highest TMS-induced MEP amplitudes was analyzed for the five central EEG channels ipsilateral to the stimulation site. We did not find any phase consistency over participants (p > 0.1). We would like to note that using a different outlier criterion (standard deviations instead of a distance measure) did lead to significant phase consistency. However, as this was not consistent across outlier criteria we do not believe this to be a robust effect.”

Considering we don’t find a robust effect, it also seems unnecessary to repeat the analysis in source space. 

3) When considering the inter-individual phase consistency related to high MEP amplitude the authors focused on the 50% amplitude of the MEP. Please justify this a priori selection.

In order to look at phase consistency across participants we need a means to estimate the phase at which the MEP is strongest (as a circular-linear correlation does not provide you the mean angle). The approach that seemed most appropriate was to split the data to extract a phase angle. The most straightforward approach would be a median split, this is why the 50% was chosen. From these top 50% MEP it was possible to estimate a phase angle and to subsequently look at phase consistency.

4) In Figure 1F and H the topographies are not very clear. Perhaps I would saturate the colour-scale and/or I would set to zero the non-significant values.

We have changed the color scheme to make it clearer. As we analyzed the data with a-priori channels we cannot differentiate values from being significant or not as this has only been quantified across the topography. We now highlight the five channels that we analyzed. 

5) - This is optional - Fecchio et al. (Plos One, 2017) showed that the EEG responses to TMS (TEPs) are different in presence/absence of MEP. Since the authors collect the EEG during TMS, It would very be interesting to test whether not only the MEPs, but also the TEPs are modulated by the phase of the ongoing activity.

We would love to perform these analyses, however during data collection we did not optimize recordings to be able to look at the TEPs. Unfortunately, in the early TEP components which are commonly analyzed in relation to MEPs we have a lot of artifacts that make it impossible to look at those components. 

MINORS

1) LINE 104 ”The EEG and EMG data analyzed for this study is part of a larger TMS study that has been analyzedand published separately (24). The data was collected during a single control session of that largerstudy, which included one block of repetitive sham TMS, but no other form of real TMS in addition to the experimental single TMS pulses included in the analysis.” This sentence is reported in the analysis section, I would report that in the procedure. I would also clearly indicate a the beginning of the Methods that these data have been collected in the context of a previous study.

We thank the reviewer for his comment. We moved the paragraph on the context of this experimental session as part of a larger study to the beginning of the Methods section and we added a brief description on the larger study setup for clarification. 

2) Please report the protocol number for the Ethical approval and indicate whether (I hope so) the participants signed any informed consent.

We added the protocol number for the approval of the Ethics Committee at Maastricht University. The signing of the informed consent is mentioned in the participant description of the Methods section.

References:

Axmacher, N., Henseler, M. M., Jensen, O., Weinreich, I., Elger, C. E., & Fell, J. (2010). Cross-frequency coupling supports multi-item working memory in the human hippocampus. Proceedings of the National Academy of Sciences, 107(7), 3228-3233. 

Canolty, R. T., Edwards, E., Dalal, S. S., Soltani, M., Nagarajan, S. S., Kirsch, H. E., . . . Knight, R. T. (2006). High gamma power is phase-locked to theta oscillations in human neocortex. science, 313(5793), 1626-1628. 

Gasser, T., Bächer, P., & Möcks, J. (1982). Transformations towards the normal distribution of broad band spectral parameters of the EEG. Electroencephalography and clinical neurophysiology, 53(1), 119-124. 

Smulders, F. T., Ten Oever, S., Donkers, F. C., Quaedflieg, C. W., & van de Ven, V. (2018). Single‐trial log transformation is optimal in frequency analysis of resting EEG alpha. European Journal of Neuroscience.

Attachment Submitted filename: Response Letter.pdf Click here for additional data file.

# Peer review: Decision Letter 1

**DOI:** 10.1371/journal.pone.0255815.r003

**Authors:** Luigi Cattaneo

26 Jul 2021

Phase and power modulations on the amplitude of TMS-induced motor evoked potentials

PONE-D-21-07587R1

Dear Dr. Ten Oever,

We’re pleased to inform you that your manuscript has been judged scientifically suitable for publication and will be formally accepted for publication once it meets all outstanding technical requirements.

Within one week, you’ll receive an e-mail detailing the required amendments. When these have been addressed, you’ll receive a formal acceptance letter and your manuscript will be scheduled for publication.

An invoice for payment will follow shortly after the formal acceptance. To ensure an efficient process, please log into Editorial Manager at http://www.editorialmanager.com/pone/, click the 'Update My Information' link at the top of the page, and double check that your user information is up-to-date. If you have any billing related questions, please contact our Author Billing department directly at authorbilling@plos.org.

If your institution or institutions have a press office, please notify them about your upcoming paper to help maximize its impact. If they’ll be preparing press materials, please inform our press team as soon as possible -- no later than 48 hours after receiving the formal acceptance. Your manuscript will remain under strict press embargo until 2 pm Eastern Time on the date of publication. For more information, please contact onepress@plos.org.

Kind regards,

Luigi Cattaneo, MD, PhD

Academic Editor

PLOS ONE

Additional Editor Comments (optional):

Reviewers' comments:

Reviewer's Responses to Questions

**Comments to the Author**

1. If the authors have adequately addressed your comments raised in a previous round of review and you feel that this manuscript is now acceptable for publication, you may indicate that here to bypass the “Comments to the Author” section, enter your conflict of interest statement in the “Confidential to Editor” section, and submit your "Accept" recommendation.

Reviewer #1: All comments have been addressed

Reviewer #2: All comments have been addressed

**********

2. Is the manuscript technically sound, and do the data support the conclusions?

The manuscript must describe a technically sound piece of scientific research with data that supports the conclusions. Experiments must have been conducted rigorously, with appropriate controls, replication, and sample sizes. The conclusions must be drawn appropriately based on the data presented. 

Reviewer #1: Yes

Reviewer #2: Yes

**********

3. Has the statistical analysis been performed appropriately and rigorously? 

Reviewer #1: Yes

Reviewer #2: (No Response)

**********

4. Have the authors made all data underlying the findings in their manuscript fully available?

The PLOS Data policy requires authors to make all data underlying the findings described in their manuscript fully available without restriction, with rare exception (please refer to the Data Availability Statement in the manuscript PDF file). The data should be provided as part of the manuscript or its supporting information, or deposited to a public repository. For example, in addition to summary statistics, the data points behind means, medians and variance measures should be available. If there are restrictions on publicly sharing data—e.g. participant privacy or use of data from a third party—those must be specified.

Reviewer #1: Yes

Reviewer #2: Yes

**********

5. Is the manuscript presented in an intelligible fashion and written in standard English?

PLOS ONE does not copyedit accepted manuscripts, so the language in submitted articles must be clear, correct, and unambiguous. Any typographical or grammatical errors should be corrected at revision, so please note any specific errors here.

Reviewer #1: Yes

Reviewer #2: Yes

**********

6. Review Comments to the Author

Please use the space provided to explain your answers to the questions above. You may also include additional comments for the author, including concerns about dual publication, research ethics, or publication ethics. (Please upload your review as an attachment if it exceeds 20,000 characters)

Reviewer #1: MINOR

line 92: "While within participant there was a systematic relation between alpha phase and MEP,...."

since this point has not been explored statistically I would avoid it. Statistical data analyses have been conducted checking the statistical consistency of the relationship between phase and MEP amplitudes across participants (and not within participants).

Reviewer #2: (No Response)

**********

7. PLOS authors have the option to publish the peer review history of their article (what does this mean?). If published, this will include your full peer review and any attached files.

If you choose “no”, your identity will remain anonymous but your review may still be made public.

**Do you want your identity to be public for this peer review?** For information about this choice, including consent withdrawal, please see our Privacy Policy.

Reviewer #1: No

Reviewer #2: No

# Editor report: Acceptance letter

**DOI:** 10.1371/journal.pone.0255815.r004

**Authors:** Luigi Cattaneo

8 Sep 2021

PONE-D-21-07587R1 

Phase and power modulations on the amplitude of TMS-induced motor evoked potentials 

Dear Dr. Ten Oever:

I'm pleased to inform you that your manuscript has been deemed suitable for publication in PLOS ONE. Congratulations! Your manuscript is now with our production department. 

If your institution or institutions have a press office, please let them know about your upcoming paper now to help maximize its impact. If they'll be preparing press materials, please inform our press team within the next 48 hours. Your manuscript will remain under strict press embargo until 2 pm Eastern Time on the date of publication. For more information please contact onepress@plos.org.

If we can help with anything else, please email us at plosone@plos.org. 

Thank you for submitting your work to PLOS ONE and supporting open access. 

Kind regards, 

PLOS ONE Editorial Office Staff

on behalf of

Dr. Luigi Cattaneo 

Academic Editor

PLOS ONE
